# Retraction: Understanding How Patients With Lumbar Radiculopathy Make Sense of and Cope With Their Symptoms

**DOI:** 10.7759/cureus.r170

**Published:** 2025-03-05

**Authors:** Pooja Samant, Poonam Tawde, Divya N Tawde

**Affiliations:** 1 Physical Therapy, Cardiff University, Cardiff, GBR; 2 Medicine, Avalon University School of Medicine, Willemstad, CUW; 3 Medicine, Chandler Regional Medical Center, Chandler, USA; 4 Health Sciences, D&D Global Co, Toronto, CAN

The article has been retracted by the Editors-in-Chief due to multiple concerns regarding methodological inconsistencies and ethical misstatements. The study includes non-qualitative references in a qualitative synthesis, with misrepresented citations that compromise the validity of the article. Additionally, the ethical considerations section contains misleading claims regarding institutional adherence and study selection.

